# Surgical Approach to Managing an Apical Periodontal Cyst Using Titanium-Activated Platelet-Rich Fibrin (T-PRF) in a Pediatric Patient: A Case Report

**DOI:** 10.7759/cureus.63887

**Published:** 2024-07-05

**Authors:** Harikishan Kanani, Rutuja Patil, Monika Khubchandani, Ramakrishna Yeluri, Rajanikanth K, Ruchika Pandey, Prachi Suroliya, Riya Goyal, Mrunali Deshkar

**Affiliations:** 1 Pediatric Dentistry, Sharad Pawar Dental College and Hospital, Datta Meghe Institute of Higher Education and Research, Wardha, IND; 2 Pedodontics and Preventive Dentistry, Sharad Pawar Dental College and Hospital, Datta Meghe Institute of Higher Education and Research, Wardha, IND; 3 Oral and Maxillofacial Surgery, Sharad Pawar Dental College and Hospital, Datta Meghe Institute of Higher Education and Research, Wardha, IND; 4 Orthodontics and Dentofacial Orthopedics, Sharad Pawar Dental College and Hospital, Datta Meghe Institute of Higher Education and Research, Wardha, IND; 5 Pediatric and Preventive Dentistry, Sharad Pawar Dental College and Hospital, Datta Meghe Institute of Higher Education and Research, Wardha, IND

**Keywords:** osseo-bone graft, pediatric surgery, dentistry, t-prf, apical periodontal cyst

## Abstract

This case report discusses the surgical management of an apical periodontal cyst in a 14-year-old male patient presenting with pain in the upper anterior jaw. The patient had a history of trauma to the upper anterior teeth, leading to the development of a radicular cyst involving teeth 11 and 12. The treatment plan included initial root canal therapy followed by surgical enucleation of the cyst, apicoectomy, and retrograde filling of the affected teeth. Titanium-activated platelet-rich fibrin (T-PRF) membranes were utilized along with an osseo-bone graft to promote healing and bone regeneration. Post-operative aesthetic rehabilitation was achieved, and the patient showed complete healing upon six months of follow-up. The case highlights the efficacy of combining endodontic therapy with surgical intervention using advanced biomaterials to manage radicular cysts in pediatric patients successfully.

## Introduction

An inflammatory odontogenic cyst is known as a radicular cyst, an apical periodontal cyst, a periapical cyst, or a dental root end cyst. The Greek word kystis, which means sac or bladder, is where the word "cyst" originates. Usually, dental caries or trauma triggers the activation and proliferation of epithelial remnants, also called cell rests of Malassez, and they are believed to be the source of the epithelium lining the lumen in specific lesions that progress to become radicular cysts [1]. This cyst is frequently discovered near the ends of the impacted tooth's roots or, when connected to an auxiliary or lateral canal, along the lateral side [2]. Jaw cysts are the most prevalent kind of cysts affecting the premolar area of the lower jaw, accounting for between 52.3% and 68% of all cases. It is more significant in the anterior part of maxillary teeth than mandibular teeth [2].

In the primary dentition, radicular cysts are uncommon, accounting for just 0.5-2.3% of all cases in both the primary and permanent dentitions [3]. For radicular cysts in the primary teeth, caries is the most common etiological cause [3]. Traumatic damage to the prominent teeth is also their cause [4]. The fourth and fifth decades are when male predominance occurs. Women may be less likely, as evidenced by the reduced incidence in this group that other workers have also seen [5].

Surgical endodontics is a proper corrective method for teeth with periapical lesions when they do not respond well to traditional root canal therapy or when orthograde treatment is not an option. It aids in maintaining the form, functioning, and aesthetics of the affected teeth and their roots after conservative instrumental, pharmaceutical, and physiotherapeutic therapy has failed [5].

Cyst management and other periapical diseases are usually treated primarily with conventional root canal therapy. Research has demonstrated that calcium hydroxide intracanal medication, a non-surgical treatment option, may effectively cure cysts and other significant periapical lesions. Periapical surgery, however, could be taken into consideration as a workable substitute in cases where traditional root canal therapy is not feasible or has failed. Enucleation and apicoectomy were shown to be the most common treatments for radicular cysts in retrospective observational research [6].

The effective treatment of a radicular cyst is described in this case report. The methods used included traditional root canal therapy, cystic enucleation, osseo-bone grafts, and titanium-activated platelet-rich fibrin (T-PRF) membranes.

## Case presentation

**Figure 1:**
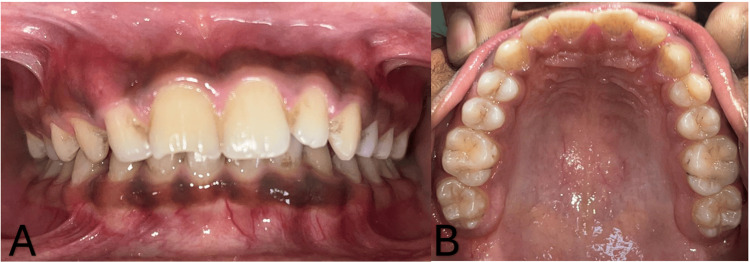
Intraoral photograph A) occlusion view B) maxillary arch

A 14-year-old male patient reported to the Department of Pediatrics and Preventive Dentistry with a chief complaint of pain in the upper front region of the jaw for one week. The patient gave a history of trauma in the upper anterior teeth, which had occurred more than four years ago due to falling from a bicycle while riding. There was no relevant past medical, family, or psychosocial history. Intraoral clinical examination revealed Ellis class IV fractures of 11 and 12 with no discoloration seen. The tests indicated that 11 (upper right central incisor) and 12 (upper right lateral incisor) had negative results from the electric and thermal pulp vitality tests, while 13 (upper right canine) showed a delayed reaction. The percussion test revealed positive teeth 11 and 12 (Figure 1).

**Figure 2:**
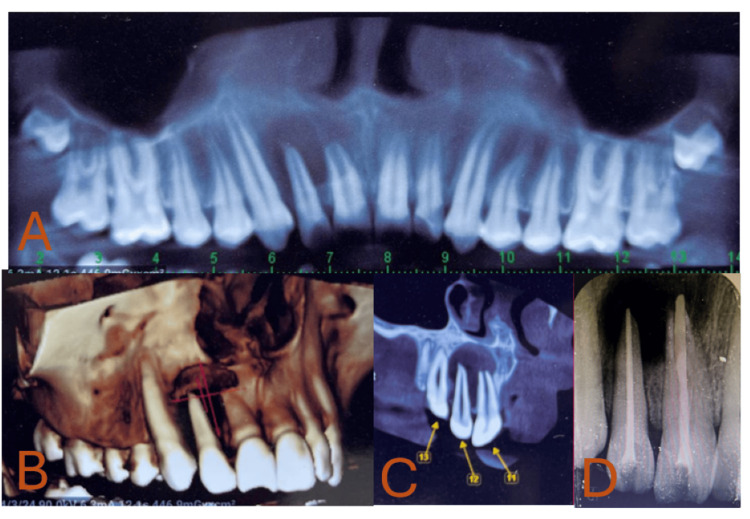
A) Unilocular radiolucency seen in CBCT; B) panoramic view; C) CBCT shows nasal floor was unbroken, and radiolucency was observed in the sagittal and coronal planes with respect to 11 and 12; D) root canal treatment done in relation to 11 and 12 CBCT: cone-beam computed tomography

A significant unilocular radiolucent lesion in the periodical region of 11 and 12, was discovered during an intraoral periapical radiograph (IOPA). Moreover, an open apex with 11 was seen. There was no cortical enlargement and unilocular radiolucency with sclerotic boundaries on the true occlusal X-ray and Orthopantomograph (OPG). A cone-beam computed tomography (CBCT) scan was performed on the patient to investigate the discovery further and determine whether the lesion was close to or included the nasal floor, given the extent of the lesion. CBCT revealed that the nasal floor was not involved, and radiolucency was observed in the sagittal and coronal planes with respect to 11 and 12. A 3D construction picture was plotted using them. An infected radicular cyst in patient's 11 and 12 was the provisional diagnosis based on the history, clinical examination, and investigation (Figure 2).

After explaining the treatment plan to the patient as well as his parents, his parent's informed consent was obtained. The rubber dam was used during the access opening procedures for patient's 11 and 12. Following the access opening, pus was discharged with 11 and 12. After assessing the working length and biomechanical preparation, intracanal calcium hydroxide medication was placed for one week. The over-obturation (two millimeters to the apex) was then finished at the following appointment. A customized cone approach was used to manage teeth 11 with an open apex (Figure 2).

**Figure 3:**
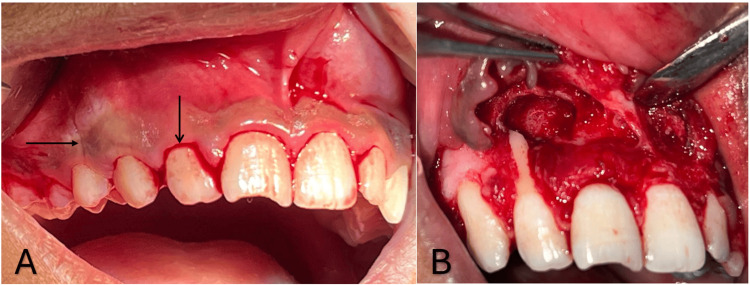
A) Horizontal and vertical incisions; B) enucleated cystic lesion

On the next appointment, the patient's surgical enucleation of the cyst, apicoectomy, and retrograde filling of the affected tooth were planned. A crevicular incision was performed in the labial areas 13 to 23 following the injection of local anesthesia. Radiographical findings revealed a significant bone defect between 11 and 12, and a full-thickness mucoperiosteal flap was reflected. After enucleating the cystic lesion, removing granulation tissue, and performing complete curettage, the lesion was sent for histological analysis. After resecting the root ends of 11 and 12, flowable light-cured glass ionomer cement (GIC) was used for the retrograde filling (Figure 3).

**Figure 4:**
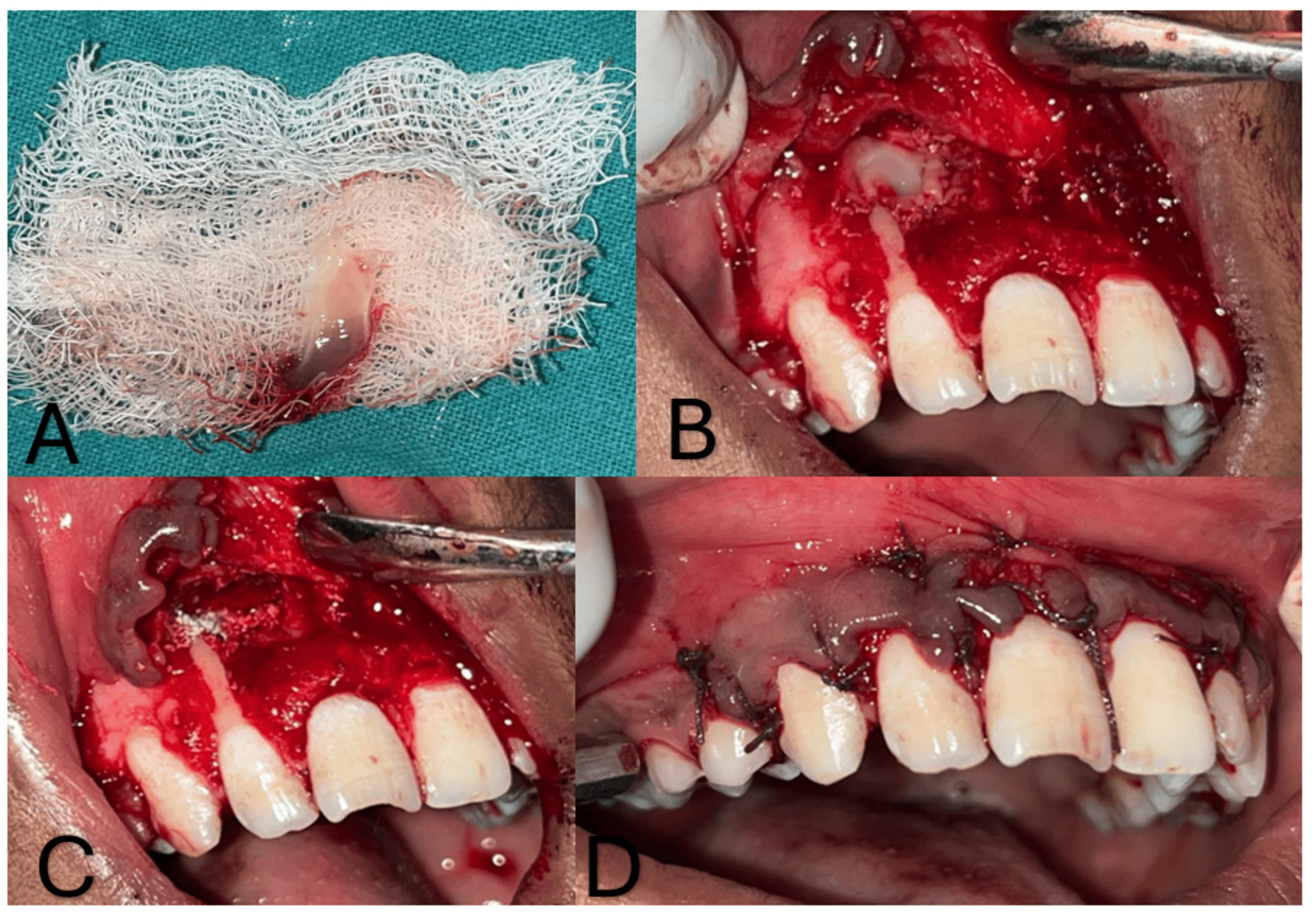
A) centrifuged T-PRF; B) T-PRF placed in cystic lesion; C) Osseo-bone graft in the defect; D) suturing done using Vicryl 4–0 T-PRF: titanium-activated platelet-rich fibrin

Two sterile 6-milliliter sterile vacutainer tubes were filled with about 5 milliliters of whole venous blood, devoid of the anticoagulant. After that, the titanium tubes were run for 10 minutes at a speed of 3000 revolutions per minute (rpm) in a centrifugal machine. Centrifuged and T-PRF was collected. After placing T-PRF and an osseo-bone graft (Advance Biotech, Chennai, India) in the defect, Vicryl 4-0 was used to close the flap. The patient received post-operative instructions and was advised to take analgesics (Ibuprofen 200 mg for five days BD) and antibiotics (Augmentin 375 mg for five days BD) (Figure 4).

**Figure 5:**
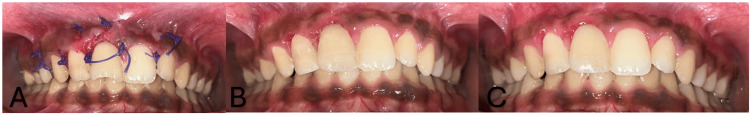
Follow-up intraoral photographs A) one week B) one month C) three months

**Figure 6:**
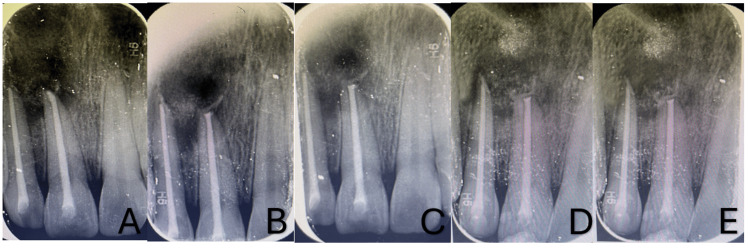
Follow-up radiographs A) baseline B) one week C) one month D) three months E) six months

An infected radicular cyst was the provisional diagnosis that was validated by the histology findings. Using tooth-colored restorative material (Composite), aesthetic rehabilitation with 11 and 12 was completed in the next session. As of now, the patient has been under follow-up for six months and showed no symptoms (Figure 5). Follow-up radiographs showed appreciable healing (Figure 6).

## Discussion

A radicular cyst is an inflammatory odontogenic cyst that develops from a proliferating Malassez cell remnant and a prolonged periapical granuloma. The bone resorbs as a result of these inflammatory lesions. They can grow dramatically and show signs of infection or neurological compression. They usually have a size range of 0.5 cm to 1.5 cm [7].

Three separate phases have been identified as part of the pathogenesis of radicular cysts namely initiation, formation, and enlargement. Radicular cysts are generally asymptomatic and are diagnosed by radiography. Still, long-standing instances may reveal an acute worsening of the cystic lesion and develop signs and symptoms such as swelling, tooth movement, and displacement of an unerupted tooth [8]. The present case also showed that pain and associated teeth are non-vital.

Clinical features and the patient's overall health influence the therapeutic option [9]. Only smaller cysts may be treated using endodontic techniques; for larger cysts, marsupialization or even enucleation or decompression is recommended [10]. Routine root canal therapy is effective in most cases of radicular cysts. Research indicates that calcium hydroxide must be used for at least two weeks to have an antibacterial effect and to decrease exudate [11]. Therefore, we used a temporary calcium hydroxide intracanal medication to treat the root canal over the course of several sessions. Then, enucleation was done as it was a large defect. One benefit of enucleation is that patients with poor compliance might benefit from early rehabilitation, meaning fewer control appointments [12]. The significant defect and potential harm to nearby structures due to the surgical operation are drawbacks. The increased risk of wound infection is another factor [13]. 

We decided to treat the radicular cysts with apical surgery because it is the gold standard approach. Moreover, this offers the advantage of permitting an excisional biopsy for histological analysis to validate the diagnosis. Histologically, nonkeratinised stratified squamous epithelium lines the inside of radicular cysts. The lining's thickness can range from 1 to 50 cell layers, making it discontinuous. Early on, a proliferative epithelial lining may exhibit arcading and a chronic solid inflammatory infiltration. The lining becomes quiescent with some differentiation as the cyst grows, resembling a stratified squamous epithelium. PMNs primarily comprise the inflammatory cell infiltration in the growing epithelium [14].

A bone graft can be used as an osteoconductive material that promotes the migration of osteoprogenitor cells, stabilizes blood clotting, and accelerates bone healing. OsseograftTM is used in this case. It is a type I collagen-based, sterile, bioresorbable bovine bone. It is made from around 250 μm-sized particles of bovine cortical bone, which are entirely replaced by host bone in 4-6 months [15].

T-PRF, the latest platelet concentrate, is used in this case. It is a three-generation platelet concentration that promotes regeneration. In addition to TPRF's capacity to remove any potential adverse effects of silica-activated PRF in glass test tubes, they enhanced fibrin meshwork, such as lengthy and dense fibrin meshwork, longer resorption times, and comparatively lower costs. For this reason, an Osseo graft and TPRF combination are used for better results [16].

## Conclusions

The case report illustrates the successful management of a radicular cyst in a pediatric patient using a combination of traditional root canal therapy, cystic enucleation, and advanced regenerative techniques, including the application of an osseo-bone graft and T-PRF. The approach effectively addressed the cystic lesion, facilitated significant bone healing, and maintained the functionality and aesthetics of the affected teeth. This comprehensive treatment highlights the importance of integrating conventional and innovative methods to achieve optimal outcomes in complex endodontic cases. The patient's favorable prognosis, evidenced by the absence of symptoms and significant healing over a one-year follow-up, underscores the efficacy of the chosen therapeutic strategies.
